# Cell-Extrinsic Priming Increases Permissiveness of CD4+ T Cells to Human Immunodeficiency Virus Infection by Increasing C–C Chemokine Receptor Type 5 Co-receptor Expression and Cellular Activation Status

**DOI:** 10.3389/fmicb.2021.763030

**Published:** 2021-11-26

**Authors:** Jesper G. Pedersen, Johanne H. Egedal, Thomas A. Packard, Karthiga Thavachelvam, Guorui Xie, Renée Marije van der Sluis, Warner C. Greene, Nadia R. Roan, Martin R. Jakobsen

**Affiliations:** ^1^Department of Biomedicine, Aarhus University, Aarhus, Denmark; ^2^Gladstone Institute of Virology, San Francisco, CA, United States; ^3^Department of Urology, University of California, San Francisco, San Francisco, CA, United States; ^4^Aarhus Institute of Advanced Studies, Aarhus University, Aarhus, Denmark

**Keywords:** human immunodeficiency virus (HIV), CD4 T cell, priming, CCR5, PD-1

## Abstract

The chemokine receptor CCR5 is expressed on multiple cell types, including macrophages, dendritic cells, and T cells, and is the major co-receptor used during HIV transmission. Using a standard αCD3/CD28 *in vitro* stimulation protocol to render CD4+ T cells from PBMCs permissive to HIV infection, we discovered that the percentage of CCR5^+^ T cells was significantly elevated in CD4+ T cells when stimulated in the presence of peripheral blood mononuclear cells (PBMCs) as compared to when stimulated as purified CD4+ T cells. This indicated that environmental factors unique to the T-PBMCs condition affect surface expression of CCR5 on CD4+ T cells. Conditioned media from αCD3/CD28-stimulated PBMCs induced CCR5 expression in cultures of unstimulated cells. Cytokine profile analysis of these media suggests IL-12 as an inducer of CCR5 expression. Mass cytometric analysis showed that stimulated T-PBMCs exhibited a uniquely activated phenotype compared to T-Pure. In line with increased CCR5 expression and activation status in stimulated T-PBMCs, CD4+ T cells from these cultures were more susceptible to infection by CCR5-tropic HIV-1 as compared with T-Pure cells. These results suggest that in order to increase *ex vivo* infection rates of blood-derived CD4+ T cells, standard stimulation protocols used in HIV infection studies should implement T-PBMCs or purified CD4+ T cells should be supplemented with IL-12.

## Introduction

Immune cells, including CD4+ T cells, express a diverse array of chemokine receptors in a regulated fashion, and the expression patterns of these receptors dictate trafficking between lymphoid tissues and the periphery. The C–C chemokine receptor type 5 (CCR5) has been extensively studied because it serves as the main co-receptor for human immunodeficiency virus type 1 (HIV-1) during sexual transmission (Alkhatib et al., 1996; Choe et al., 1996; Deng et al., 1996; Doranz et al., 1996; Dragic et al., 1996). Its expression associates with susceptibility of cells to HIV infection *in vitro* (Wu et al., 1997; Platt et al., 1998; Tuttle et al., 1998) and viral load *in vivo* (Marmor et al., 2000). Furthermore, CCR5 antagonists reduce HIV-1 replication and a delta-32 mutation in the CCR5 receptor renders CD4+ T cells resistant to CCR5-tropic HIV infection (Dean et al., 1996; Liu et al., 1996; Samson et al., 1996b; Dorr et al., 2005; Fätkenheuer et al., 2005; Gulick et al., 2008).

CCR5 is also involved in many fundamental inflammatory processes such as recruiting lymphocytes to inflammatory sites, protection against influenza and West Nile virus, and has recently been shown to affect cognition enhancement in mice (Lim and Murphy, 2011; Pozo et al., 2015; Zhou et al., 2016). CCR5 is highly expressed on a fraction of memory CD4+ T cells and macrophages, and directs immune cells to inflammatory sites via MIP1a, MIP1b, RANTES, and MCP2 signaling (Combadiere et al., 1996; Raport et al., 1996; Samson et al., 1996a; Gong et al., 1998; Nibbs et al., 1999). Expression levels of CCR5 display considerable inter-individual variation (Moore, 1997), but is uniformly induced upon T cell receptor (TCR) stimulation. Additionally, certain cytokines have been suggested to regulate CCR5 expression on CD4+ T cells. Long term stimulation with Interleukin (IL)-2 increases CCR5 expression (Bleul et al., 1997; Wang et al., 1999; Yang et al., 2001) and IL-12 upregulates CCR5 expression on TCR-stimulated human and murine CD4+ T cells (Iwasaki et al., 2001; Mukai et al., 2001; Yang et al., 2001). Other cytokines such as tumor necrosis factor alpha (TNF-α) and interferon-gamma (IFN-γ) have also been found to promote upregulation of CCR5 on CD4+ T cells and monocytes, whereas IL-10 has been found to downregulate CCR5 expression (Hariharan et al., 1999; Patterson et al., 1999).

In 1989, Engleman et al. found that T cell activation was required for HIV-1 to efficiently infect blood-derived CD4+ T cells *in vitro* (Gowda et al., 1989; Lifson and Engleman, 1989). They found that treatment with phytohaemagglutinin (PHA) or αCD3 monoclonal antibodies (mAbs) rendered cells permissive to viral integration. Over the years, various stimulation protocols have been explored, but the standard cocktails currently used to render PBMCs permissive to HIV infection include stimulation with PHA or αCD3/αCD28 in the presence of IL-2 (Gowda et al., 1989; Fortin et al., 2001). Protocols have been implemented where the CD4+ T cells are activated either as purified CD4+ T cells, or in the context of bulk PBMCs which includes paracrine effects of CD8 T cells, B cells, NK cells, monocytes and dendritic cells. Although CD4+ T cell activation is routinely used for *in vitro* HIV infection studies, to our knowledge no study has directly compared the stimulation of isolated CD4+ T cells with the stimulation of CD4+ T cells in the context of PBMCs. We discovered that CD4+ T cells activated in the context of a PBMC culture have increased CCR5 expression as compared to CD4+ T cells activated as a purified population. Furthermore, CD4+ T cells activated within PBMCs display a more activated phenotype with high expression levels of the cellular activation markers CD25, CD69, CD38, and immune checkpoint molecule protein death 1 (PD-1), and are significantly more susceptible to infection by CCR5-tropic HIV.

## Results

### C–C Chemokine Receptor Type 5 Expression Is Dependent on Activation Stimuli and Environmental Factors

**FIGURE 1:**
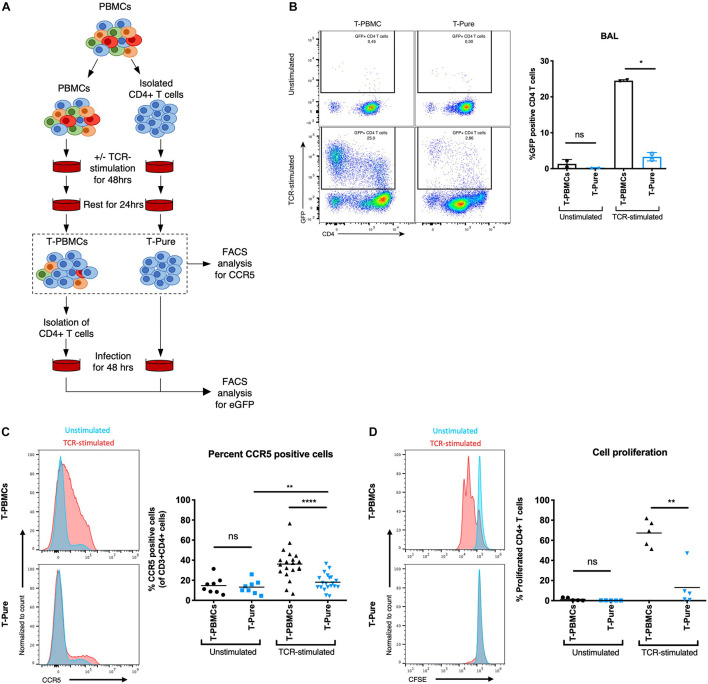
CD4+ T cells from T- peripheral blood mononuclear cells (PBMCs) express elevated levels of cell-surface C-C chemokine receptor type 5 (CCR5) relative to those from T-Pure cultures. **(A)** Illustration of experimental setup to compare CD4+ T cells stimulated under different conditions. CD4+ T cells were mock-treated or stimulated with plate-bound αCD3(OKT3)/CD28(CD28.2) + IL-2 (termed TCR-stimulation) for 48 h either within the bulk PBMC population (termed T-PBMCs) or as a purified population of CD4+ T cells (termed T-Pure). Following T cell receptor (TCR)-activation, the cells were washed to remove antibodies and rested for another 24 h in the absence of stimulation. Cells treated in this manner were used for infection studies or analyzed by flow cytometry for CCR5 expression levels and proliferative capacity **(B–D)**. For infection studies CD4+ T cells were purified from the bulk T-PBMC population before infection was performed. **(B)** Cells treated as described in panel **(A)** were infected for 48 h with 100 ng p24/well of a CCR5-tropic Bal HIV-1 encoding an GFP reporter. Shown on the left are FACS plots showing the frequency of GFP+ cells. Shown on the right are quantitation of the data from two donors. **(C)** Cells treated as described in panel **(A)** were analyzed by flow cytometry for cell-surface levels of CCR5. Shown on the left are representative histograms from one donor showing cells expressing elevated levels of CCR5 amongst the T-PBMCs (top) vs. T-Pure cells (bottom) under unstimulated (blue) and TCR-stimulated (red) conditions. The graph on the right shows the proportions of CCR5+ cells under each of the culture conditions (*n* = 8 donors for unstimulated and 20 donors for TCR-stimulated conditions). **(D)** To quantify cell proliferation, cells were stained with CFSE prior to the treatment conditions described in panel **(A)**. The proportion of CFSE (low) cells, corresponding to those that have undergone proliferation, is shown on representative FACS plots on the left. Shown on the right are quantitation of the data from *n* = 5 donors. Paired students *t*-test was used for all analyses. ns, not significant; *P < 0.05; **P < 0.01; ****P < 0.0001.

We first investigated the permissiveness of CD4+ T cells to infection with CCR5-tropic HIV-1 BAL harboring a GFP reporter (BAL-GFP) under different stimulation conditions. CD4+ T cells were stimulated with plate-bound αCD3/CD28 + IL-2 (hereafter referred to as TCR-stimulated) in the context of bulk PBMCs (hereafter referred to as T-PBMCs) or as purified CD4+ T cells (hereafter referred to as T-Pure), isolated by negative selection from the same HIV-negative donors (Figure 1A). After stimulation of T-PBMC, CD4+ T cells were isolated and equal numbers of CD4+ T cells were cultured with BAL-GFP for 48 h. Subsequently, cells were harvested and flow cytometry was used to analyse the frequency of GFP + cells within the CD3+ CD8- cell population, which includes CD4+ T cells that have downregulated cell-surface CD4 due to HIV infection (Ren et al., 2014; Tavares et al., 2017). As expected, infection was inefficient in unstimulated T-PBMCs and unstimulated T-Pure, consistent with the notion that resting PBMCs are poorly permissive to infection (Gowda et al., 1989; Figure 1B). In the TCR-stimulated cell cultures, significantly higher infection rates were observed in T-PBMCs as compared to the T-Pure cells (Figure 1B).

We then investigated whether this difference in permissiveness was associated with cell-surface expression levels of CCR5. In the absence of TCR-stimulation, T-PBMCs and T-Pure displayed comparable frequencies of CCR5+ cells while TCR stimulation resulted in a significant increase in the frequency of CCR5+ cells for T-PBMCs as compared to T-Pure (Figure 1C and Supplementary Figure 1). The same trend was observed for PHA-stimulated cells (Supplementary Figure 2). We also observed a trend toward increased CD4 expression on HIV BAL exposed TCR-stimulated T-PBMCs compared with T-Pure cells (Supplementary Figure 3A). Similar observations were found for non-HIV-BAL exposed cells, demonstrating that TCR-stimulation increased CD4 expression on T-PBMCs compared with T-Pure cells (Supplementary Figure 3B).

A hallmark of the response of CD4+ T cells to stimulation via the TCR is cellular proliferation (Au-Yeung et al., 2014). Interestingly, proliferation as monitored by CFSE dilution was more prominent in the T-PBMCs condition (mean 67% proliferated cells) as compared to the T-Pure condition (mean 13% proliferated cells) (Figure 1D). Furthermore, CD4+ T cell blasting, a measure of CD4+ T cell activation, was more pronounced in the T-PBMCs stimulation condition (Supplementary Figure 4). Taken together, these results demonstrate that CCR5 expression is highly dependent on the context of cellular stimulation.

### Peripheral Blood Mononuclear Cell-Secreted Factors Increase C–C Chemokine Receptor Type 5 Expression on CD4+ T Cell

**FIGURE 2:**
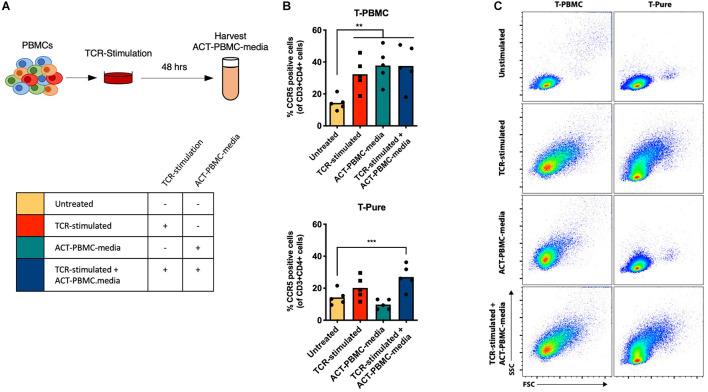
Transfer of TCR-stimulated T-PBMC media increases the frequency of CCR5+ CD4+ T cells. **(A)** Schematic representation of experimental set-up. PBMCs were TCR-stimulated for 48 h, and then cell culture supernatant (ACT-PBMC-media) was collected for transfer to new cell cultures of T-PBMCs and T-Pure cells. T-PBMCs and T-pure cells were treated as described in Figure 1A except that ACT-PBMC-media was added at a 1:1 v/v ratio during the stimulation as indicated in the scheme. **(B)** Graph showing the frequencies of CCR5+ CD4+ T cells (*n* = 5) under the experimental design described in panel **(A)**. **(C)** Flow cytometry plots showing T cell blasting by cells treated as described in panel **(A)**. Results are gated on live, singlet CD3+ CD4+ cells and are representative of data from one of five donors. Bars indicate mean values and each dot represent an individual donor. One-way ANOVA comparing untreated sample to every other sample. **P < 0.01; ***P < 0.001.

To determine how non-CD4+ T cells within the PBMC population contribute to CD4+ T cell activation, we examined whether cell-to-cell contact was required by evaluating the effects of cell culture media from PBMCs that were TCR-stimulated for 48 h and then depleted of cells (ACT-PBMC-media) (Figure 2A). ACT-PBMC-media was added to T-PBMCs and T-Pure cells in the presence or absence of additional TCR stimulation (Figure 2B). TCR stimulation of T-PBMCs increased the frequency of CD4+ CCR5+ T cells, as expected (Figure 2B, top graph). Addition of ACT-PBMC-media to unstimulated T-PBMCs also increased the frequency of CD4+ CCR5+ T cells as compared to untreated cells, while addition of ACT-PBMC-media to TCR-stimulated T-PBMCs did not further increase the frequency of CD4+ CCR5+ T cells as compared to TCR-stimulation or ACT-PBMC-media alone (Figure 2B, top graph). In contrast, neither TCR-stimulation nor addition of ACT-PBMC-media increased the frequency of CD4+ CCR5+ T cells in T-Pure cells compared with untreated T-Pure cells (Figure 2B, bottom graph). However, combining TCR-stimulation with ACT-PBMC-media significantly increased the frequency of CD4+ CCR5+ T cells as compared to untreated T-Pure cells (Figure 2B, bottom graph), albeit not to the same extent as observed for ACT-PBMC-media treated T-PBMCs. To confirm that soluble factors in ACT-PBMC-media resulting from TCR stimulation were responsible, we tested as a negative control the effects of cell culture medium from unstimulated PBMC (mock-PBMC-media) and found that it did not increase CCR5 expression on T-PBMCs and T-Pure (Supplementary Figure 5). Interestingly, T-PBMCs blasted upon addition of ACT-PBMC-media, whereas no blasting was observed for T-Pure cells treated with ACT-PBMC-media (Figure 2C). Taken together, the data demonstrate that media from TCR-stimulated PBMCs contains soluble factors that increases the frequency of CCR5+ CD4+ T cells in unstimulated T-PBMCs and TCR-stimulated T-Pure cells but not unstimulated T-Pure cells.

### Interleukin-12 Increases C–C Chemokine Receptor Type 5 Expression on Stimulated Pure CD4+ T Cells

**FIGURE 3:**
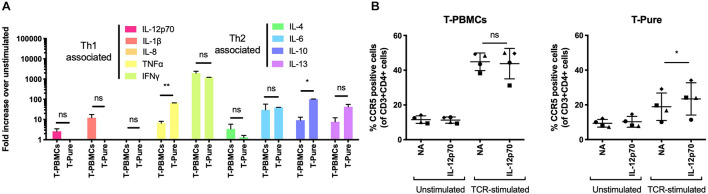
Interleukin-12 enhances CCR5 expression on CD4+ T cells from T-Pure cultures. **(A)** T-PBMCs and T-Pure cells were TCR-stimulated or left unstimulated for 72 h. Cell culture supernatants were then harvested and analyzed for expression of 9 cytokines by the MSD V-Plex proinflammatory panel 1 kit. Cytokine expression within TCR-stimulated cells is represented as fold-increase over unstimulated cells and grouped based on T helper 1 (Th1)- and T helper 2 (Th2)-associated cytokines. (*n* = 2 donors). Paired students *t*-test, *P < 0.05, **P < 0.01, ns, not significant. **(B)** T-PBMCs (left) and T-Pure cells (right) were left unstimulated or TCR-stimulated for 48 h. Cells were then washed to remove antibodies, cultured with or without 0.25 ng/ml recombinant human IL-12p70 for an additional 48 h and the frequency of CCR5+ CD4+ T cells was analyzed by flow cytometry. Lines indicate mean and symbols represent individual donors (*n* = 4). Paired students *t*-test, *P < 0.05, ns, not significant; NA, no addition.

Pro-inflammatory cytokines, including IL-2 and IL-12, have been shown to increase CCR5 expression on CD4+ T cells (Yang et al., 2001). To assess whether these or other cytokines may be responsible for the observed upregulation of CCR5, we evaluated the cytokine composition of media from TCR-stimulated T-PBMCs and TCR-stimulated T-Pure cells. We used the MSD V-Plex proinflammatory cytokine panel to measure the concentrations of the following 9 cytokines: IL-1b, IL-4, IL-6, IL-8, IL-10, IL-12, IL-13, TNFα, and IFNγ. IL-2 was excluded from the analysis as it was added to the medium during cultivation. Cytokine levels in TCR-stimulated samples were normalized to unstimulated samples. IL-12, IL-1β, IFNγ, and IL-4 were present at higher concentrations in supernatants from TCR-stimulated PBMCs as compared to supernatants from TCR-stimulated T-Pure cells (Figure 3A). In contrast, IL-10, IL-13, and TNFα were present at higher levels in supernatant from T-Pure cells as compared to T-PBMCs, with TNFα and IL-10 being significantly elevated (Figure 3A).

Given that IL-12 levels were elevated in supernatant from PBMCs compared to T-Pure cells, and has been reported to induce CCR5 expression (Iwasaki et al., 2001; Mukai et al., 2001; Yang et al., 2001), we tested how IL-12 stimulation affected the frequency of CCR5+ CD4+ T cells in the different cell culture conditions. Stimulation with IL-12 (0.25 ng/ml) did not affect the number of CCR5+ cells in T-PBMCs, in the absence or presence of TCR-stimulation (Figure 3B). No effect on the frequency of CCR5+ cells was observed when IL-12 was administered to unstimulated T-Pure cells. However, IL-12 treatment of TCR-stimulated T-Pure cells significantly elevated the number of CCR5+ cells (Figure 3B), consistent with previous reports (Iwasaki et al., 2001; Mukai et al., 2001; Yang et al., 2001). In conclusion, our data demonstrates that IL-12 stimulation of TCR-stimulated T-Pure cells enhances the frequency of CCR5+ cells.

### T Cell Receptor-Stimulated T-Peripheral Blood Mononuclear Cells Are Highly Activated and Phenotypically Distinct From T Cell Receptor-Stimulated T-Pure Cells

**FIGURE 4:**
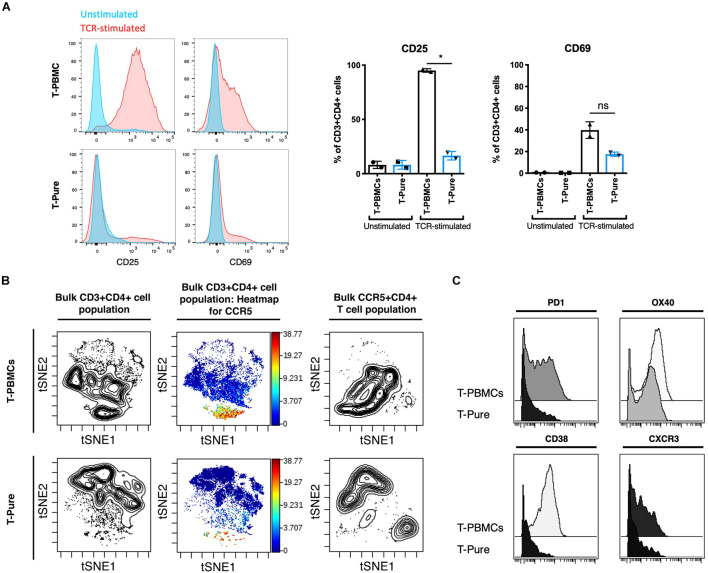
T- peripheral blood mononuclear cells are more highly activated and phenotypically distinct from T-Pure cells. **(A)** Representative flow cytometry histogram showing expression of extracellular activation markers CD25 and CD69 on T-PBMCs and T-Pure cells that were TCR-stimulated or left unstimulated for 48 h followed by 24 h of rest without stimuli as described in Figure 1A. Graphs on the right show mean frequencies of CD25+ and CD69+ CD4+ T cells (*n* = 2 donors). **(B)** T-PBMCs and T-Pure cells were analyzed using a 35-parameter CyTOF phenotyping panel, and results are presented as t-SNE plots. The first two contour plots on the left demonstrate that T-PBMCs (top) are different from those from T-Pure cells (bottom), as demonstrated by the cells residing in distinct regions of the tSNE plot. The same plots are then shown in heatmap fashion colored by expression levels of CCR5. Note the higher proportion of CCR5-expressing cells under the T-PBMCs culture conditions. The contour plots on the right correspond to a new tSNE analysis conducted on the CD4+ CCR5+ cells from the same donor sample. The CCR5+ CD4+ T cells from the T-PBMCs condition (top) reside in a distinct region of the tSNE plot relative to CCR5+ CD4+ T cells form the T-Pure condition (bottom), suggesting that the phenotype of CCR5+ CD4+ T cells are different under the two stimulation conditions. Two additional donors analyzed in a similar fashion are shown in Supplementary Figure 6A. **(C)** The CD4+ CCR5+ T cells from the T-PBMCs and T-Pure cultures were analyzed for expression of the activation markers PD1, OX40, CD38, and the T helper cell 1 marker CXCR3. Two additional donors analyzed in a similar fashion are shown in Supplementary Figure 6B. Statistical analysis was performed using a Paired students *t*-test. *P < 0.05. ns, not significant.

Thus far, our data suggest that T-PBMCs exhibit higher frequencies of CCR5+ cells as compared to T-Pure cells, and this associates with higher infection rates. Because productive HIV infection is not only dependent on receptor/co-receptor usage, but also on the activation state of the cell, we next assessed whether the increased HIV permissiveness of the CD4+ T cells in the T-PBMCs cultures is associated with an increased state of cellular activation. Expression of the T cell activation markers CD25 and CD69 was analyzed by flow cytometric analysis. Upon TCR stimulation, CD25 expression was increased significantly on T-PBMCs as compared to T-Pure cells (Figure 4A). CD69 expression was also increased on T-PBMCs compared with T-Pure cells, although the effect was not statistically significant (Figure 4A). To further characterize the phenotypic differences between T-PBMCs and T-Pure cells, we utilized a 35-parameter mass cytometry panel that includes multiple markers of T cell activation (Supplementary Table 1). All samples were analyzed on a CyTOF2 instrument and live, intact CD3+ CD4+ T cells were exported for further analysis (Supplementary Figure 6) by the dimensional reduction method t-distributed stochastic neighbor embedding (tSNE), which allows visualization of high-dimensional data on a 2D plot. We first compared by t-SNE CD4+ T cells from T-PBMCs and T-Pure cultures. Contour visualization of the data suggested that CD4+ T cells in the two cultures were phenotypically distinct as revealed by different patterns observed in the t-SNE plots (Figure 4B, plots on left and Supplementary Figure 7A). We then visualized expression levels of CCR5 in heatmap fashion on these tSNE plots. Among CD4+ T cells, there was a distinct population of cells expressing high levels of CCR5 that was more prominent in the T-PBMC as compared to the T-Pure cells (Figure 4B and Supplementary Figure 7A). This is in line with our previous findings that CCR5+ CD4+ T cells are more frequent in T-PBMCs as compared to T-Pure cells. To determine whether the phenotypic properties of the CD4+ T cells expressing high levels of CCR5 were different under the T-PBMC vs T-Pure conditions, a new t-SNE analysis was then performed on gated CD4+ CCR5+ cells (Figure 4B and Supplementary Figure 7A). The CD4+ CCR5+ cells from T-PBMCs cultures resided in a region of the tSNE plot distinct from the region occupied by CD4+ CCR5+ cells from the T-Pure cultures, suggesting that not only are the frequencies of CCR5-expressing cells higher under T-PBMCs culture conditions, but that these cells are also phenotypically distinct from CCR5-expressing cells under T-Pure culture conditions.

To understand what was driving the phenotypic differences between the two populations of CD4+ CCR5+ cells, we then surveyed the expression of various markers on these cells (Supplementary Figure 8). Interestingly, CCR5+ CD4+ T cells from T-PBMC cultures expressed higher levels of the activation markers PD1, OX40 and CD38 as compared to CCR5+ CD4+ T cells from T-Pure cultures (Figure 4C and Supplementary Figure 7B). The cells from T-PBMCs also expressed higher levels of the Th1 marker CXCR3 (Loetscher et al., 1998; Figure 4C and Supplementary Figure 7B). These findings suggest that CD4+ T cells stimulated as T-PBMCs differentiate into a more Th1-like cell population, whereas CD4+ T cells stimulated as T-Pure cells differentiate more into Th2-like cells.

### Infection With C–C Chemokine Receptor Type 5-Tropic Human Immunodeficiency Virus Type 1 Is Increased in T- Peripheral Blood Mononuclear Cells

**FIGURE 5:**
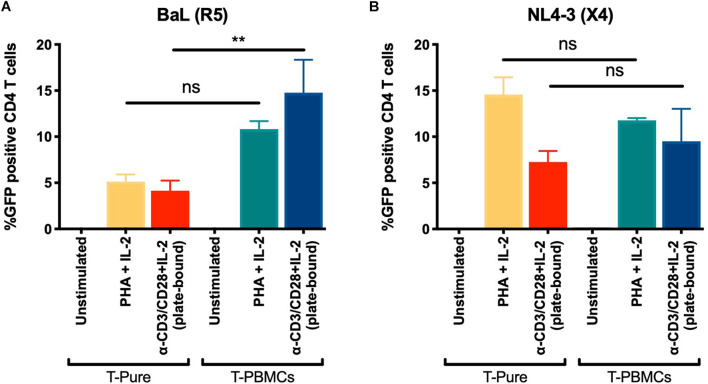
Increased infection of T-PBMCs with an CCR5 tropic HIV-1. T-PBMCs and T-Pure cells were prepared as described in Figure 1B and infected with BaL (CCR5-tropic) **(A)** or NL4-3 (CXCR4-tropic) **(B)** GFP reporter viruses at a concentration of (100 ng/well p24). Two days later, the frequencies of infected (i.e., GFP+) CD3+ CD8- T cells was quantitated by flow cytometry. Bars correspond to mean ± SD (*n* = 2 donors, each in triplicates). Statistical significance was calculated using One-way ANOVA with Tukey correction for multiple comparison. **P < 0.01. ns, not significant.

Our data demonstrate that TCR stimulation of CD4+ T cells within the context of T-PBMC gives rise to CD4+ T cells that express higher levels of CCR5 and that the CCR5+ CD4+ T cells display a more activated phenotype. CCR5 expression levels and activation state may independently affect infection rates; the former by promoting entry of CCR5-tropic HIV, and the latter by increasing the permissiveness of CD4+ T cells to HIV infection, which can affect infection rates of both CXCR4- and CCR5-tropic HIV. To assess the role of entry vs. activation in our observed phenotypes, we compared infection rates by CCR5- and CXCR4-tropic HIV. CD4+ T cells from T-PBMCs and T-Pure cultures were infected with the CCR5-tropic BaL-GFP strain or the CXCR4-tropic HIV-1 strain NL4-3 expressing GFP (NL4-3-GFP) for 48 h. For comparison, unstimulated and PHA + IL-2 stimulated cells were infected in parallel cultures. While CD4+ T cells from T-PBMCs cultures were infected by BaL more efficiently than CD4+ T cells from T-Pure cultures as expected (Figure 5A), no difference was observed in infection rates when NL4-3-GFP was instead used (Figure 5B). Of note, CXCR4 expression did not change across culture and stimulation conditions (Supplementary Figure 9). These results suggest that the increased infection rates were primarily driven by changes in CCR5 expression and not by the differential activation state of the CD4+ T cells cultured under the two conditions.

## Discussion

In this study we investigated how the environmental context of culturing CD4+ T cells affects the expression of CCR5 and susceptibility to infection by HIV-1. We demonstrate that CCR5 expression on CD4+ T cells is greatly dependent on the context in which the CD4+ T cells are stimulated. CD4+ T cells stimulated within a mixed culture of PBMCs (T-PBMCs) display markedly higher frequencies of CCR5+ cells as compared with CD4+ T cells stimulated in isolation (T-Pure). This increased CCR5 expression is driven by cytokines produced by the PBMC population, including IL-12. In addition to increased CCR5 expression, T-PBMCs also display a more activated phenotype, defined by elevated expression of the activation markers CD25, CD69, PD-1, OX40, and CD38. However, this difference in activation state may not be an important determinant of increased susceptibility of these cells to HIV infection, since infection rates by CCR5-tropic HIV, but not CXCR4-tropic HIV, are enhanced under the T-PBMC stimulation condition. Instead, the data suggest that the differential infection rates observed are due primarily to altered CCR5 expression levels. We notice that our CyTOF and FACS data demonstrated increased CD4 expression on T-PBMCs compared with T-Pure cells, further emphasizing the vast phenotypical differences induced by the two culture conditions. As CD4 is important for HIV entry and infection, this could also contribute to the increased infection rates by CCR5-tropic HIV seen for T-PBMCs. However, if CD4 expression was the limiting factor for HIV infection in this setting, differential infection rates between T-PBMCs and T-Pure cells when exposed to CXCR4-tropic HIV would be expected. As this is not the case, we believe that CD4 expression is not the primary reason for the observed differential infection rates, although further investigations are needed to confirm this.

Chemokine receptors are known for their important role during T cell trafficking and their expression is known to be regulated by T cell activation state and cytokines. Previous studies have demonstrated that IL-12 increases CCR5 expression on CD4+ T cells (Iwasaki et al., 2001; Mukai et al., 2001; Yang et al., 2001). We found that IL-12 was present in the supernatant from T-PBMCs, but not in supernatant from T-Pure cells upon TCR-stimulation, which may explain why T-PBMCs have increased frequency of CCR5+ CD4+ T cells. Supporting this idea was our demonstration that both ACT-PBMC-media and recombinant IL-12 increased the frequency of CCR5+ CD4+ T cells in TCR-stimulated T-Pure cells, but not in unstimulated T-Pure cells. These findings are supported by the fact that the IL-12 receptor is expressed on CD4+ T cells after TCR-stimulation but not before, explaining why TCR-stimulated CD4+ T cells, but not unstimulated CD4+ T cells, respond to ACT-PBMC-media and IL-12 stimulation (Rogge et al., 1997; Szabo et al., 1997; Igarashi et al., 1998; Tomura et al., 1998; Yang et al., 2001). Despite increasing levels of CCR5+ cells in T-Pure cells upon IL-12 or ACT-PBMC-media treatment in combination with TCR-stimulation, the levels did not reach the levels observed in T-PBMCs, suggesting that multiple cytokines in combination may regulate CCR5 expression. Such cytokines could include IL-2, IFNγ, and TNFα, which have been shown to increase CCR5 expression on CD4+ T cells, or IL-10, which has been shown to downregulate its expression (Patterson et al., 1999). Further investigations are needed to clarify how CCR5 expression is modulated by cytokines, including IL-10 which was differentially expressed between T-PBMCs and T-Pure cells. It is possible that cell-to-cell contact may also contribute to regulating CCR5 expression. Thus, further studies are warranted to better understand the mechanisms underlying the regulation of CCR5 expression in CD4 T cells activated in the context of bulk PBMCs. Such studies should include determining to what extent different PBMC subsets, particularly antigen-presenting cells including monocytes, are important for this effect, as well as assessing the signaling pathways involved. Finally, as HIV permissiveness is known to be strongly depend on levels of CCR5 surface expression (Platt et al., 1998; Mulampaka and Dixit, 2011), it is also likely that increasing its expression on T-Pure cells by stimulation with IL-12 or other cytokines may increase their ability to be infected with CCR5-tropic HIV. Future studies should directly address the effect of exogenous IL-12 stimulation on permissivity of T-Pure cells to infection.

We further explored if the stimulation context affected CD4+ T cell activation. We demonstrated that TCR-stimulated T-PBMCs markedly increased expression of CD25, CD69, PD1, CD38, and OX40, as compared to TCR-stimulated T-Pure cells, indicating that T-PBMCs are more activated than T-Pure cells. T-PBMCs also had a higher expression of CXCR3 than T-Pure cells. Given that CXCR3 is a Th1 marker, our data suggest that T-PBMCs display a more Th1-like phenotype. These data are consistent with another reports concluding that Th1 CD4+ T cells express high levels of CCR5, whereas Th2 CD4+ T cells express only low amount of CCR5 (Gosselin et al., 2009). However, although CD4+ T cells from T-PBMCs exhibited an activation phenotype associated with high susceptibility, our data demonstrates that infection rates by X4-tropic HIV are similar in T-PBMCs and T-Pure suggesting that increased CCR5 expression rather than increased CD4+ T cell activation is driving the high infection rates by R5-tropic HIV.

*In vivo*, CD4+ T cells are activated in a highly complex and controlled environment involving multiple signals, including signals between CD4+ T cells and antigen presenting cells (APCs). CD4+ T cells are activated through detection of antigen presented by APCs, in a manner influenced by cytokine milieu and co-stimulatory signals (Gasteiger et al., 2016). During *in vitro* activation of isolated CD4+ T cells, the interaction between APCs and CD4+ T cells is often mimicked in an antigen-agnostic manner through the use of PHA or functional antibodies targeting CD3 and CD28. However, the absence of APCs may affect the activation process as several essential signaling molecules are missing. Indeed, our data support this model. Here, we report that *in vitro* activation of CD4+ T cells in the context of other immune cells present in PBMCs results in more robust activation, as well as phenotypic and functional differences in the activated cells. This kind of activation may be more similar to the *in vivo* situation, thereby making the *in vitro* model for studying HIV infections more comparable to *in vivo* situations. Further validation of this model and its comparability to *in vivo* situations is warranted.

In summary, TCR-stimulation of CD4+ T cells within PBMCs as compared to in isolation leads to increased CCR5 expression and activation mediated by the local cytokine milieu within the PBMC population. These cells are more permissive to infection with R5-tropic HIV-1, underscoring the importance of which activation protocol being used for *in vitro* HIV infection assays.

## Materials and Methods

### Primary Cells

Peripheral blood mononuclear cells (PBMCs) were isolated by Ficoll-Hypaque density gradient centrifugation from buffy coats obtained from healthy donors at Skejby University Hospital, Aarhus, Denmark. CD4+ T cells were isolated by negative selection using the Easysep^TM^ Human CD4^+^ T cell enrichment kit (STEMCELL Technologies, #19052) according to the manufacturer’s protocol. Cells were cultured in RPMI-1640 (Sigma Aldrich, R0883) supplemented with 10% heat-inactivated fetal calf serum (iFCS) (Sigma Aldrich, F7524), 1% L-Glutamine (Gibco^®^ life Technologies), 100 Units (U)/ml penicillin and 100 μg/ml streptomycin (Gibco^®^ life technologies) (referred to as complete RPMI) at a density of 2 × 10^6^ cells/ml. Cells were cultured at 37°C, 95% humidity and 5% CO_2_. Where indicated, cells were stimulated using either plate-bound anti-CD3 (clone: OKT3, eBioscience, 16-0037-85,1 μg/ml) + plate-bound CD28 (clone: CD28.2, eBioscience, 16-0289-85, 1 μg/ml) + recombinant human IL-2 (rhIL-2, PeproTech, 200-02, 40 U/ml) or PHA (Sigma Aldrich, L1668, 5 or 10 μg/ml) + rhIL-2 (40 U/ml). Cells were stimulated for 48 h and subsequently rested for 24 or 48 h in complete RPMI supplemented with rhIL-2 (40 U/ml) but without antibodies or PHA.

### Cell Proliferation Assay

Cell proliferation was analyzed by measuring the consecutive dilution of Carboxyfluorescein succinimidyl ester (CFSE) occurring at each round of cell division. A single cell suspension of 10 mio cells in 1 ml complete RPMI was prepared and stained with CFSE (eBioscience, 65-0850-84) for 5 min at a concentration of 5 μM and subsequently washed three times with PBS + 5% iFCS. Then cells were cultured for 48 h with or without stimulation with plate-bound anti-CD3/CD28 + rhIL-2 followed by 24 h of rest, as described above. The fraction of proliferated cells, as measured by CFSE dilution, were analyzed by flow cytometry.

### ACT-PBMC-Media and Mock-Peripheral Blood Mononuclear Cells-Media

ACT-PBMC-media was produced by stimulating PBMCs with plate-bound anti-CD3 (1 μg/ml) and plate-bound anti-CD28 (1 μg/ml) for 48 h in complete RPMI supplemented with rhIL-2 (40 U/ml). Cell culture media was then harvested, centrifuged, and the supernatant was collected and filtered through a 0.2 μm filter. ACT-PBMC-media containing cytokines and other factors secreted by PBMCs was used in a 1:1 (v/v) ratio with complete RPMI. Mock-PBMC-media was produced similar to ACT-PBMC-media, except that the PBMCs were not stimulated with anti-CD3 and anti-CD28.

### Interleukin-12 Stimulation

For IL-12 stimulation, T-PBMCs and T-Pure cells were stimulated with plate-bound anti-CD3 (1 μg/ml) + plate-bound CD28 (1 μg/ml) + rhIL-2 (40 U/ml) for 48 h and rested for 48 h without stimuli. During the 48 h rest period, cells were stimulated with recombinant human IL-12p70 (0.25 ng/ml) (PeproTech, 200-12).

### Flow Cytometry

A total of 4 × 10^5^ cells per well were stained with antibodies against CD3 (APC-H7, BD pharmigen, cat# 560176), CD4 (PC-7, Beckman Coulter, cat# 737660), CD8 (BV650, BD horizon, cat# 563821), CCR5 (BV421, Biolegend, cat# 359118, clone: J418F1), CD25 (APC, Biolegend, cat# 302610, clone: BC96), CD69 (Alexa700, Biolegend, cat# 310922, clone: FN50), and zombie aqua (BV510, Biolegend, cat# 423102) as a viability marker. Compensation matrices were established using OneComp ebeads (eBioscience, 01-1111-42) and Arc reactive beads and Arc negative beads (Molecular Probes Life Technologies). Fluorescence intensity was measured by a NovoCyte flow cytometer (ACEA Bioscience). Data were analyzed by FlowJo software (version 10.4.0). Median fluorescence intensity (MFI) was obtained using FlowJo.

### CyTOF

CyTOF antibody conjugation and staining analysis were performed similar to methods previously described (Cavrois et al., 2017). Antibodies requiring in-house conjugation to metal isotopes were conjugated using X8 antibody labeling kits (Fluidigm). All metals were purchased from Fluidigm, except for 155Gd and 157Gd which were purchased from Trace Sciences. Conjugation was conducted according to manufacturer’s instructions. After the final wash, conjugated antibodies were eluted in a total of 100 μl, quantitated for protein content by Nanodrop 2000c, and diluted 1:1 with PBS-based Antibody Stabilizer (Boca Scientific) supplemented with 0.05% sodium azide, and stored at 4°C until ready for use in CyTOF staining.

On the day of staining, approximately 6 × 10^6^ cells/sample were processed in Nunc^TM^ 96 DeepWell^TM^ polystyrene plates (Thermo Fisher). After blocking the cells for 15 min at 4°C with sera from mouse (Thermo Fisher), rat (Thermo Fisher), and human (AB serum, Sigma-Aldrich), cells were washed 2× with 800 μl CyFACS buffer, consisting of metal contaminant-free PBS (Rockland) supplemented with 0.1% bovine serum albumin and 0.1% sodium azide. Cells were then stained for 45 min at 4°C with the cocktail of primary antibodies in a total volume of 100 μl/well. Cells were then washed 3× with CyFACS buffer and treated for 30 min at 4°C with 1.7 μg/ml 139In-DOTA maleimide (Macrocyclics) as a live/dead marker. Cells were then washed two more times with CyFACS buffer and fixed overnight with 2% PFA (diluted from 16% stock purchased from Electron Microscopy Sciences) in contaminant-free PBS (Rockland). The next day, cells were washed 2× with Permeabilization Buffer (eBioscience), and permeabilized for an additional 45 min at 4°C. After a subsequent wash with CyFACS, cells were treated for 20 min at 25°C with 250 nM Cell-ID^TM^ Intercalator-Ir (Fluidigm). Samples were then washed two more times with CyFACS, once with PBS (Rockland), and three times with pure water. Immediately prior to sample acquisition, samples were diluted to 6.2 × 10^5^/ml in a 1:10 dilution of EQ^TM^ calibration beads (Fluidigm). Samples were acquired at a rate of 400–800 events/sec on an upgraded CyTOF2 instrument (Fluidigm) at the UCSF Parnassus Flow Core. Prior to export, the CyTOF data were normalized to EQ^TM^ calibration beads and imported into Flowjo (Treestar) and Cytobank for population gating and data analysis. Events corresponding to T cells and the indicated subsets of T cells were extracted following sequential gating to identify these cells based on DNA content, viability, and cell length, and expression of the indicated markers.

### Virus Production and Infection Assay

The R5-tropic BaL-GFP and X4-tropic NL4-3-GFP HIV reporter viruses used in this study were produced as previously described (Neidleman et al., 2017).

For infection assays, T-PBMCs and T-Pure cells were unstimulated or TCR-stimulated as described above. CD4+ T cells were purified from the T-PBMCs and then CD4+ purified T-PBMCs and T-Pure cells (200,000 cells/well) were seeded in 96-well flat-bottom plates. Cells were infected with BaL-GFP or NL4-3-GFP (100 ng/well p24^Gag^ in 200 μ l volume) for 48 h and then harvested for flow cytometry analysis.

### Cytokine Analysis by Meso Scale Discovery

T-Peripheral blood mononuclear cells and T-pure cells were unstimulated or TCR-stimulated for 72 h as described above. Subsequently, cell culture supernatant was harvested and analyzed for the presence of IL-1b, IL-2, IL-4, IL-6, IL-8, IL-10, IL-12, IL-13, TNFα, and IFNγ, using the V-plex pro-inflammatory panel 1 human kit (Meso scale discovery) according to manufactures protocol. Cytokines were measured in the unit pg/ml. IL-2 was excluded from the analysis, as rhIL-2 was supplemented to the culture media.

### Statistics

Statistical significance was calculated using GraphPad prism version 8. Paired Students *t*-test was used for comparison of mean values between two groups, whereas unpaired one-way ANOVA with Tukey correction was used for comparison of mean values between three or more groups. *P*-values < 0.05 were considered statistical significant.

## Data Availability Statement

The original contributions presented in the study are included in the article/Supplementary Material, further inquiries can be directed to the corresponding authors.

## Author Contributions

JP, JE, TP, RS, KT, and GX performed the experiments. JP, JE, TP, NR, and MJ conceived and designed the experiments. JP, NR, and MJ wrote the manuscript. All authors contributed to the article and approved the submitted version.

## Conflict of Interest

The authors declare that the research was conducted in the absence of any commercial or financial relationships that could be construed as a potential conflict of interest.

## Publisher’s Note

All claims expressed in this article are solely those of the authors and do not necessarily represent those of their affiliated organizations, or those of the publisher, the editors and the reviewers. Any product that may be evaluated in this article, or claim that may be made by its manufacturer, is not guaranteed or endorsed by the publisher.
