# Supplementary material for: Cell-Extrinsic Priming Increases Permissiveness of CD4+ T Cells to Human Immunodeficiency Virus Infection by Increasing C–C Chemokine Receptor Type 5 Co-receptor Expression and Cellular Activation Status
Source: Front Microbiol. 2021 Nov 26;12:763030. doi: 10.3389/fmicb.2021.763030 (PMC8661899; doi:10.3389/fmicb.2021.763030)
Supplement: Supplementary file 1 [file Presentation_1.PPTX]

## Slide 1
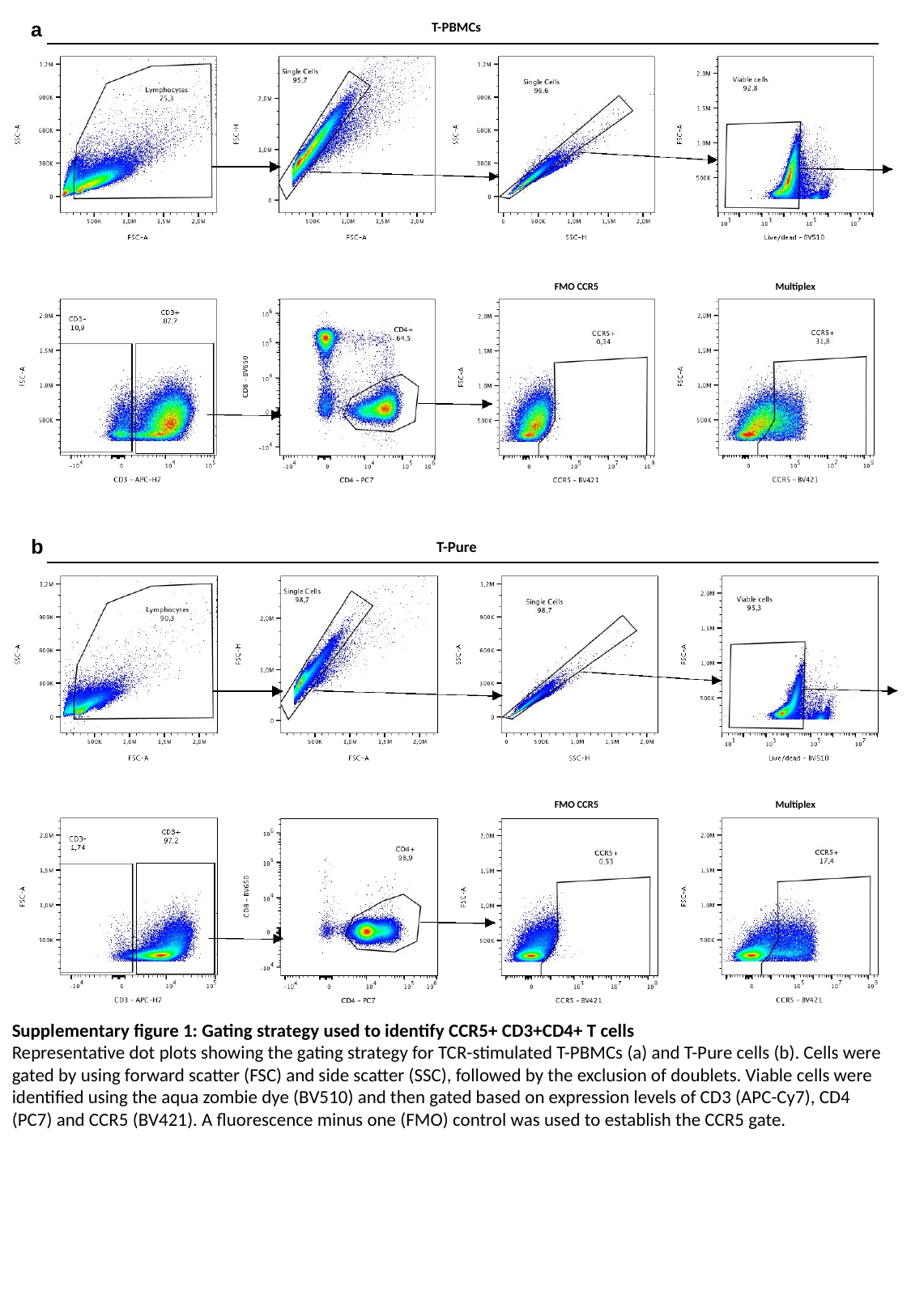

a
T-PBMCs
FMO CCR5
Multiplex
b
T-Pure
FMO CCR5
Multiplex
Supplementary figure 1: Gating strategy used to identify CCR5+ CD3+CD4+ T cells
Representative dot plots showing the gating strategy for TCR-stimulated T-PBMCs (a) and T-Pure cells (b). Cells were gated by using forward scatter (FSC) and side scatter (SSC), followed by the exclusion of doublets. Viable cells were identified using the aqua zombie dye (BV510) and then gated based on expression levels of CD3 (APC-Cy7), CD4 (PC7) and CCR5 (BV421). A fluorescence minus one (FMO) control was used to establish the CCR5 gate.

## Slide 2
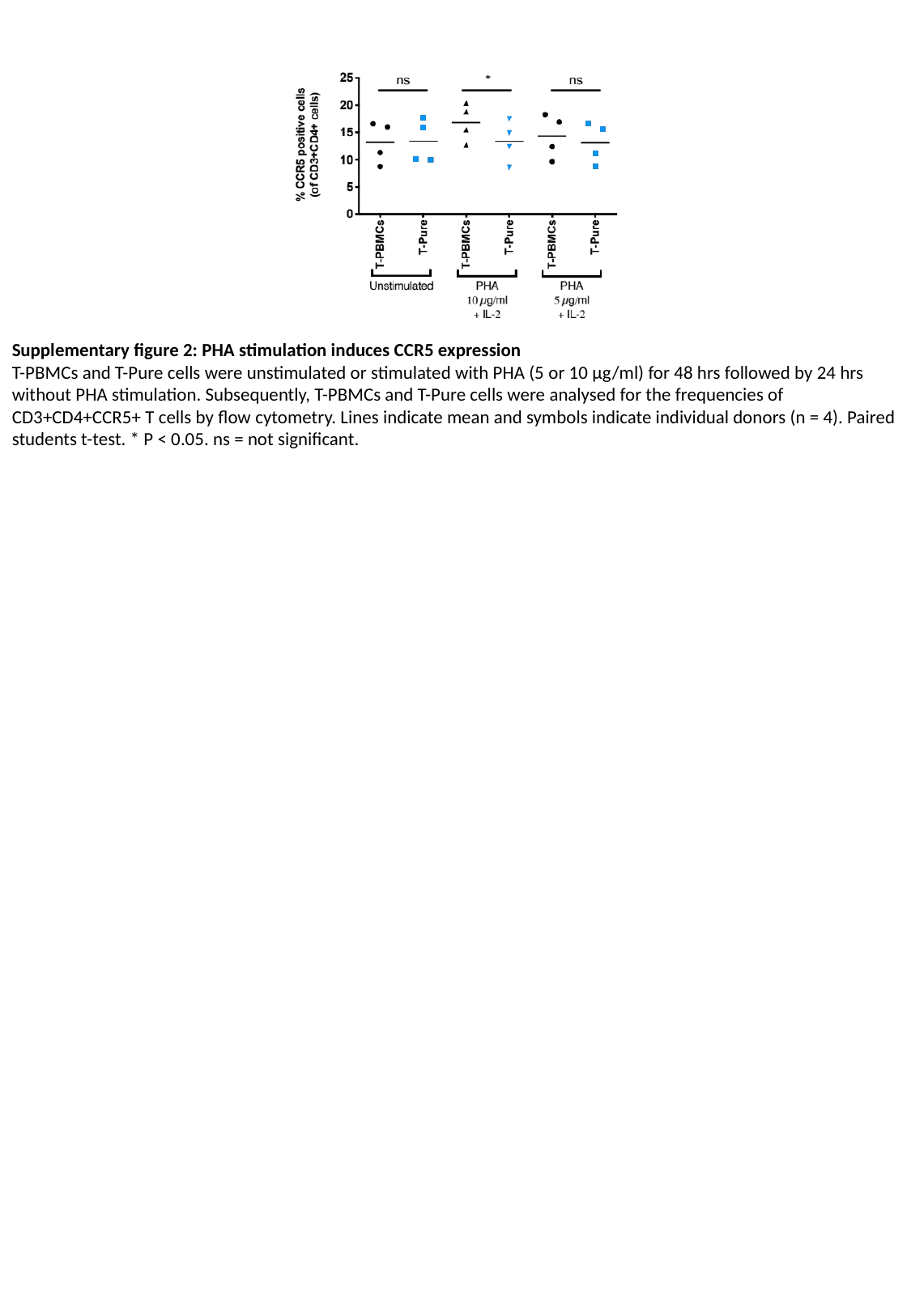

Supplementary figure 2: PHA stimulation induces CCR5 expression
T-PBMCs and T-Pure cells were unstimulated or stimulated with PHA (5 or 10 µg/ml) for 48 hrs followed by 24 hrs without PHA stimulation. Subsequently, T-PBMCs and T-Pure cells were analysed for the frequencies of CD3+CD4+CCR5+ T cells by flow cytometry. Lines indicate mean and symbols indicate individual donors (n = 4). Paired students t-test. * P < 0.05. ns = not significant.

## Slide 3
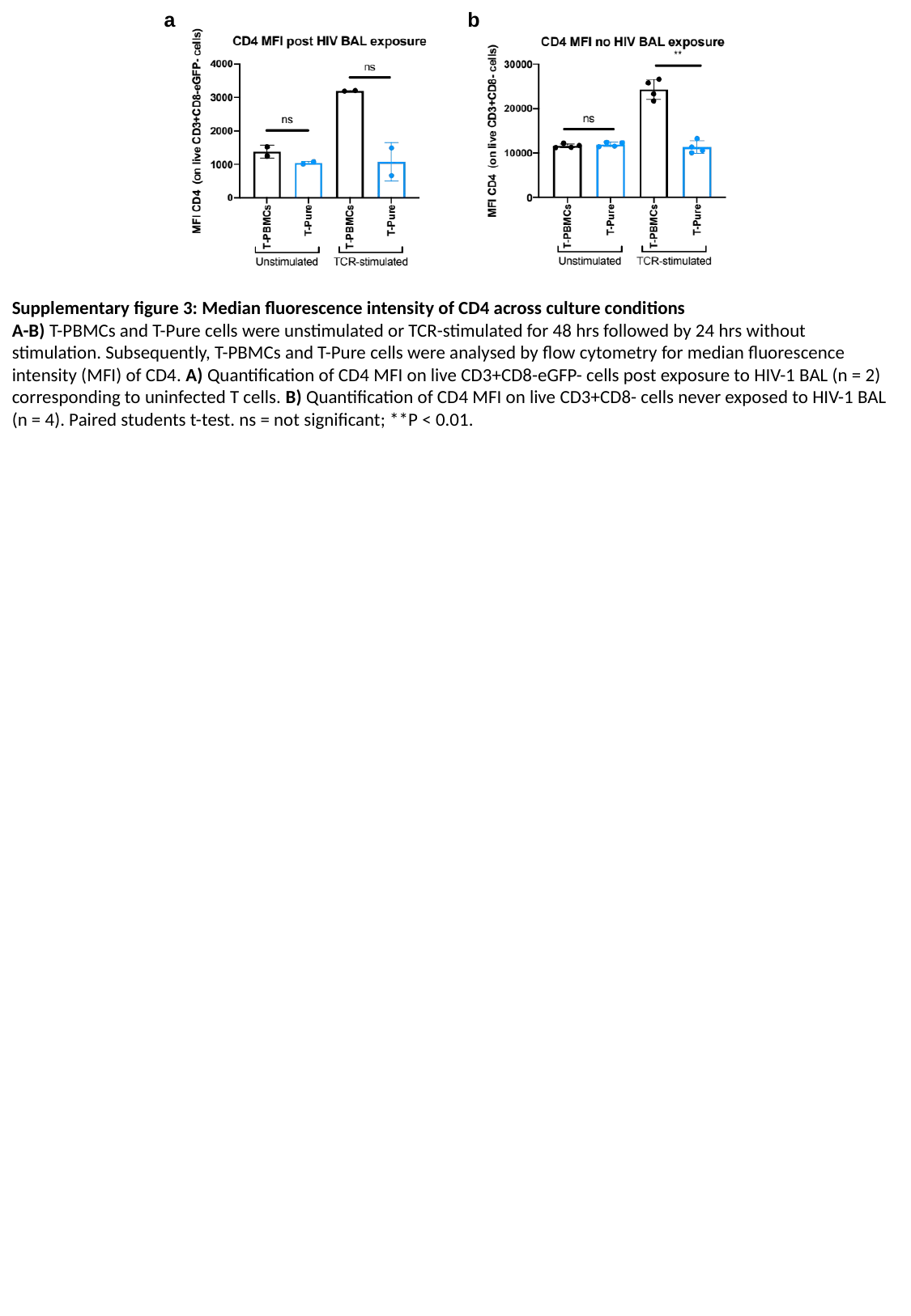

a
b
Supplementary figure 3: Median fluorescence intensity of CD4 across culture conditions
A-B) T-PBMCs and T-Pure cells were unstimulated or TCR-stimulated for 48 hrs followed by 24 hrs without stimulation. Subsequently, T-PBMCs and T-Pure cells were analysed by flow cytometry for median fluorescence intensity (MFI) of CD4. A) Quantification of CD4 MFI on live CD3+CD8-eGFP- cells post exposure to HIV-1 BAL (n = 2) corresponding to uninfected T cells. B) Quantification of CD4 MFI on live CD3+CD8- cells never exposed to HIV-1 BAL (n = 4). Paired students t-test. ns = not significant; **P < 0.01.

## Slide 4
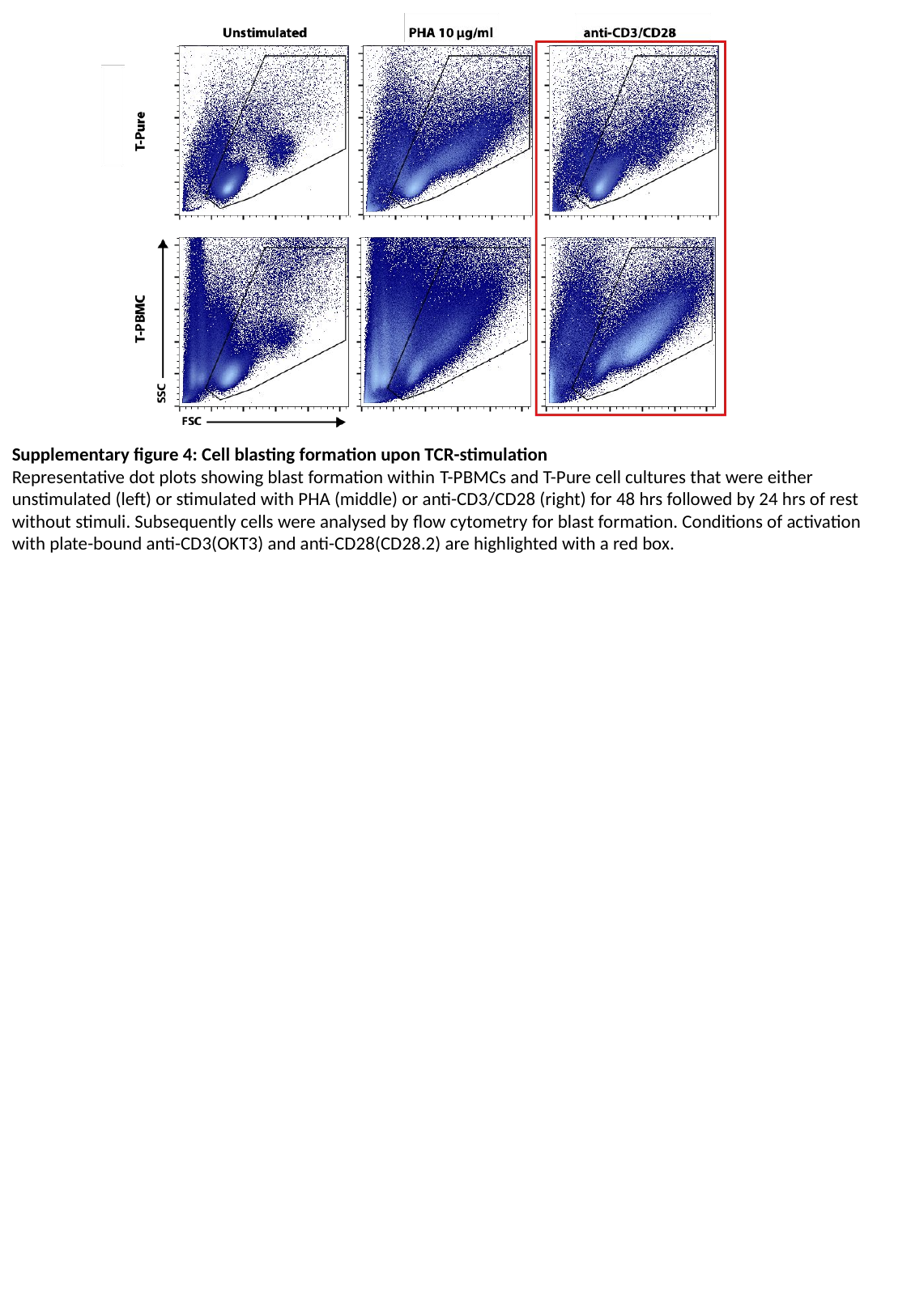

Supplementary figure 4: Cell blasting formation upon TCR-stimulation
Representative dot plots showing blast formation within T-PBMCs and T-Pure cell cultures that were either unstimulated (left) or stimulated with PHA (middle) or anti-CD3/CD28 (right) for 48 hrs followed by 24 hrs of rest without stimuli. Subsequently cells were analysed by flow cytometry for blast formation. Conditions of activation with plate-bound anti-CD3(OKT3) and anti-CD28(CD28.2) are highlighted with a red box.

## Slide 5
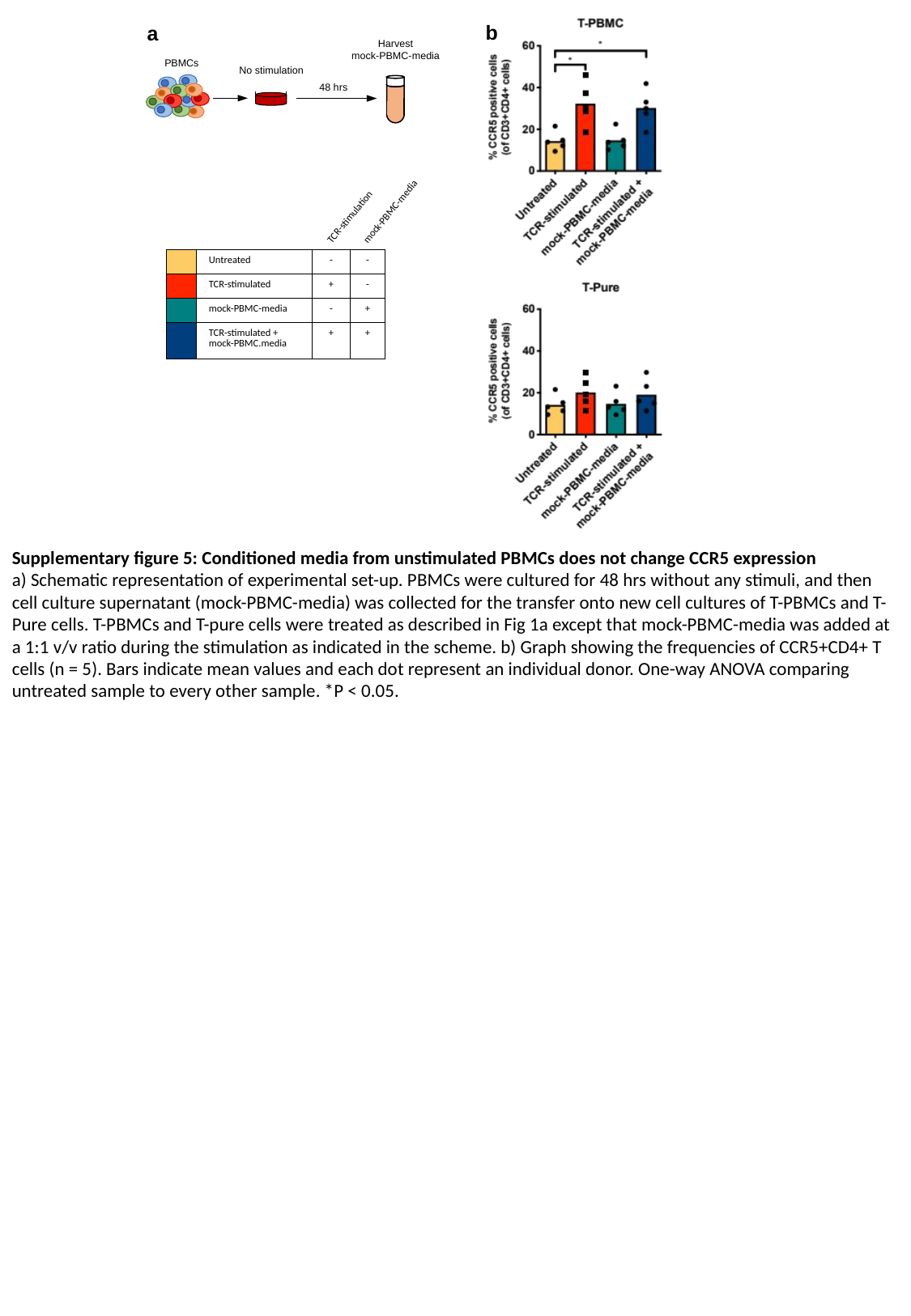

b
a
Harvest
mock-PBMC-media
PBMCs
No stimulation
48 hrs
TCR-stimulation
mock-PBMC-media
| | Untreated | - | - |
| --- | --- | --- | --- |
| | TCR-stimulated | + | - |
| | mock-PBMC-media | - | + |
| | TCR-stimulated + mock-PBMC.media | + | + |
Supplementary figure 5: Conditioned media from unstimulated PBMCs does not change CCR5 expression
a) Schematic representation of experimental set-up. PBMCs were cultured for 48 hrs without any stimuli, and then cell culture supernatant (mock-PBMC-media) was collected for the transfer onto new cell cultures of T-PBMCs and T-Pure cells. T-PBMCs and T-pure cells were treated as described in Fig 1a except that mock-PBMC-media was added at a 1:1 v/v ratio during the stimulation as indicated in the scheme. b) Graph showing the frequencies of CCR5+CD4+ T cells (n = 5). Bars indicate mean values and each dot represent an individual donor. One-way ANOVA comparing untreated sample to every other sample. *P < 0.05.

## Slide 6
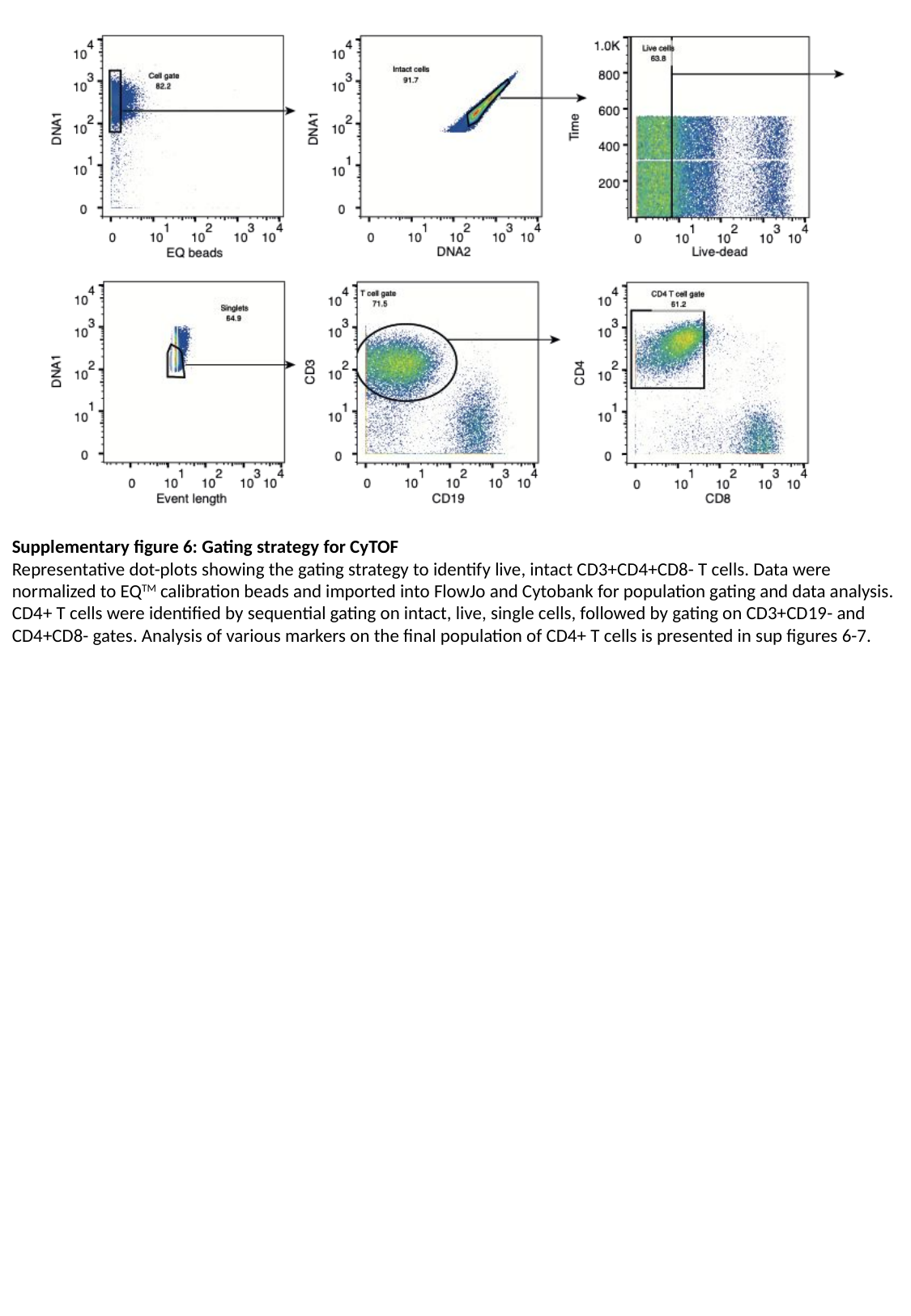

Supplementary figure 6: Gating strategy for CyTOF
Representative dot-plots showing the gating strategy to identify live, intact CD3+CD4+CD8- T cells. Data were normalized to EQTM calibration beads and imported into FlowJo and Cytobank for population gating and data analysis. CD4+ T cells were identified by sequential gating on intact, live, single cells, followed by gating on CD3+CD19- and CD4+CD8- gates. Analysis of various markers on the final population of CD4+ T cells is presented in sup figures 6-7.

## Slide 7
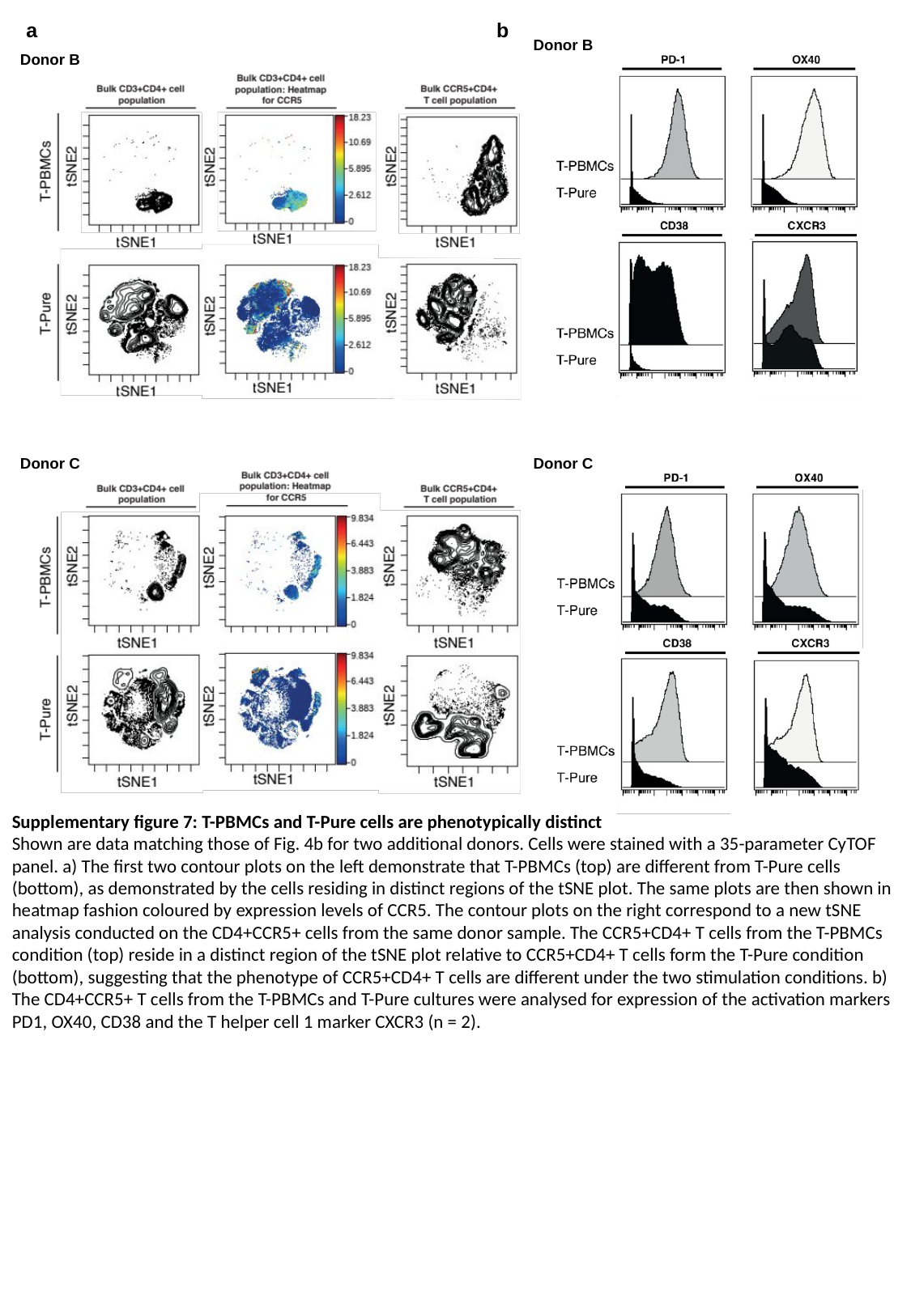

b
a
Donor B
Donor B
Donor C
Donor C
Supplementary figure 7: T-PBMCs and T-Pure cells are phenotypically distinct
Shown are data matching those of Fig. 4b for two additional donors. Cells were stained with a 35-parameter CyTOF panel. a) The first two contour plots on the left demonstrate that T-PBMCs (top) are different from T-Pure cells (bottom), as demonstrated by the cells residing in distinct regions of the tSNE plot. The same plots are then shown in heatmap fashion coloured by expression levels of CCR5. The contour plots on the right correspond to a new tSNE analysis conducted on the CD4+CCR5+ cells from the same donor sample. The CCR5+CD4+ T cells from the T-PBMCs condition (top) reside in a distinct region of the tSNE plot relative to CCR5+CD4+ T cells form the T-Pure condition (bottom), suggesting that the phenotype of CCR5+CD4+ T cells are different under the two stimulation conditions. b) The CD4+CCR5+ T cells from the T-PBMCs and T-Pure cultures were analysed for expression of the activation markers PD1, OX40, CD38 and the T helper cell 1 marker CXCR3 (n = 2).

## Slide 8
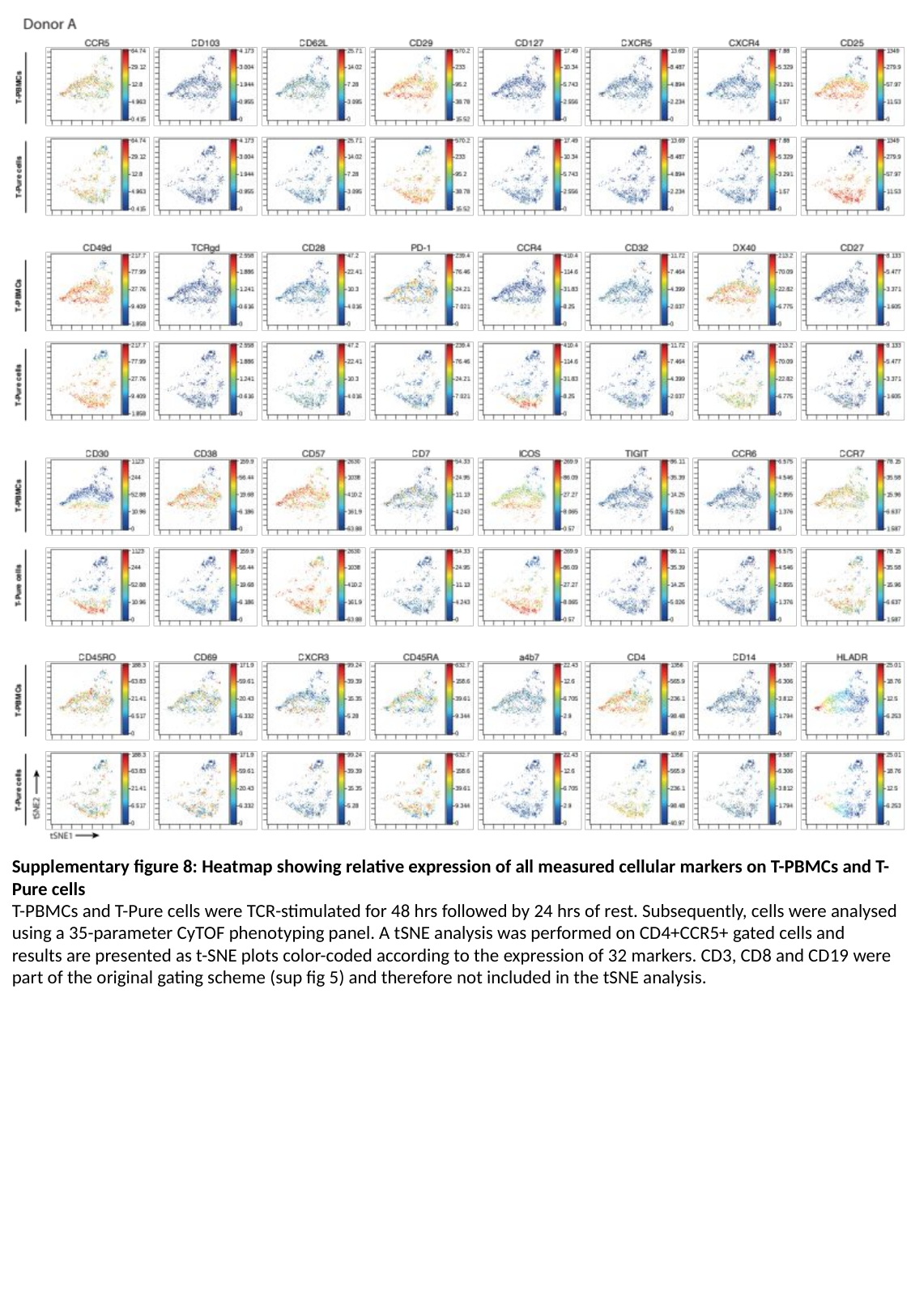

Supplementary figure 8: Heatmap showing relative expression of all measured cellular markers on T-PBMCs and T-Pure cells
T-PBMCs and T-Pure cells were TCR-stimulated for 48 hrs followed by 24 hrs of rest. Subsequently, cells were analysed using a 35-parameter CyTOF phenotyping panel. A tSNE analysis was performed on CD4+CCR5+ gated cells and results are presented as t-SNE plots color-coded according to the expression of 32 markers. CD3, CD8 and CD19 were part of the original gating scheme (sup fig 5) and therefore not included in the tSNE analysis.

## Slide 9
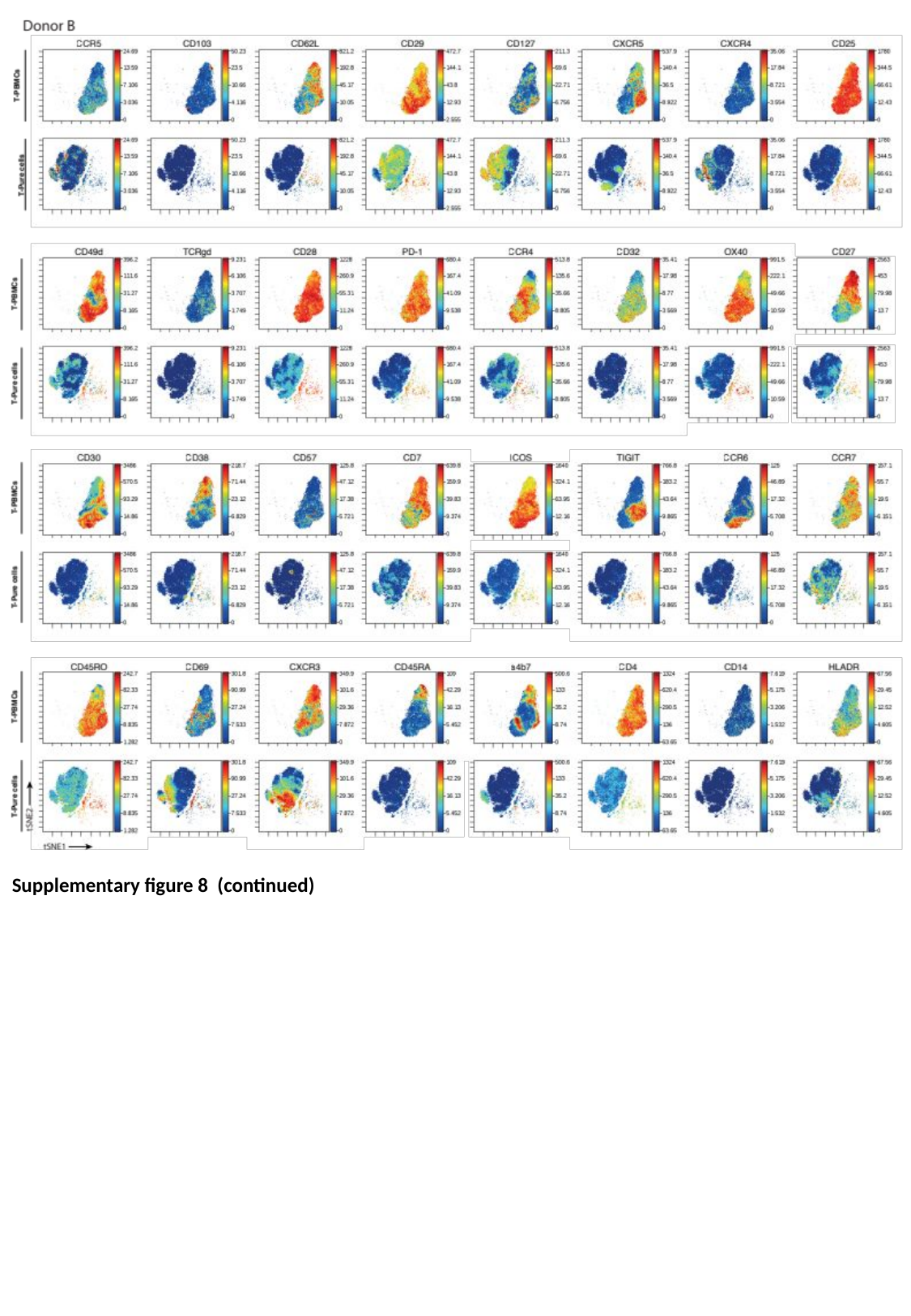

Supplementary figure 8 (continued)

## Slide 10
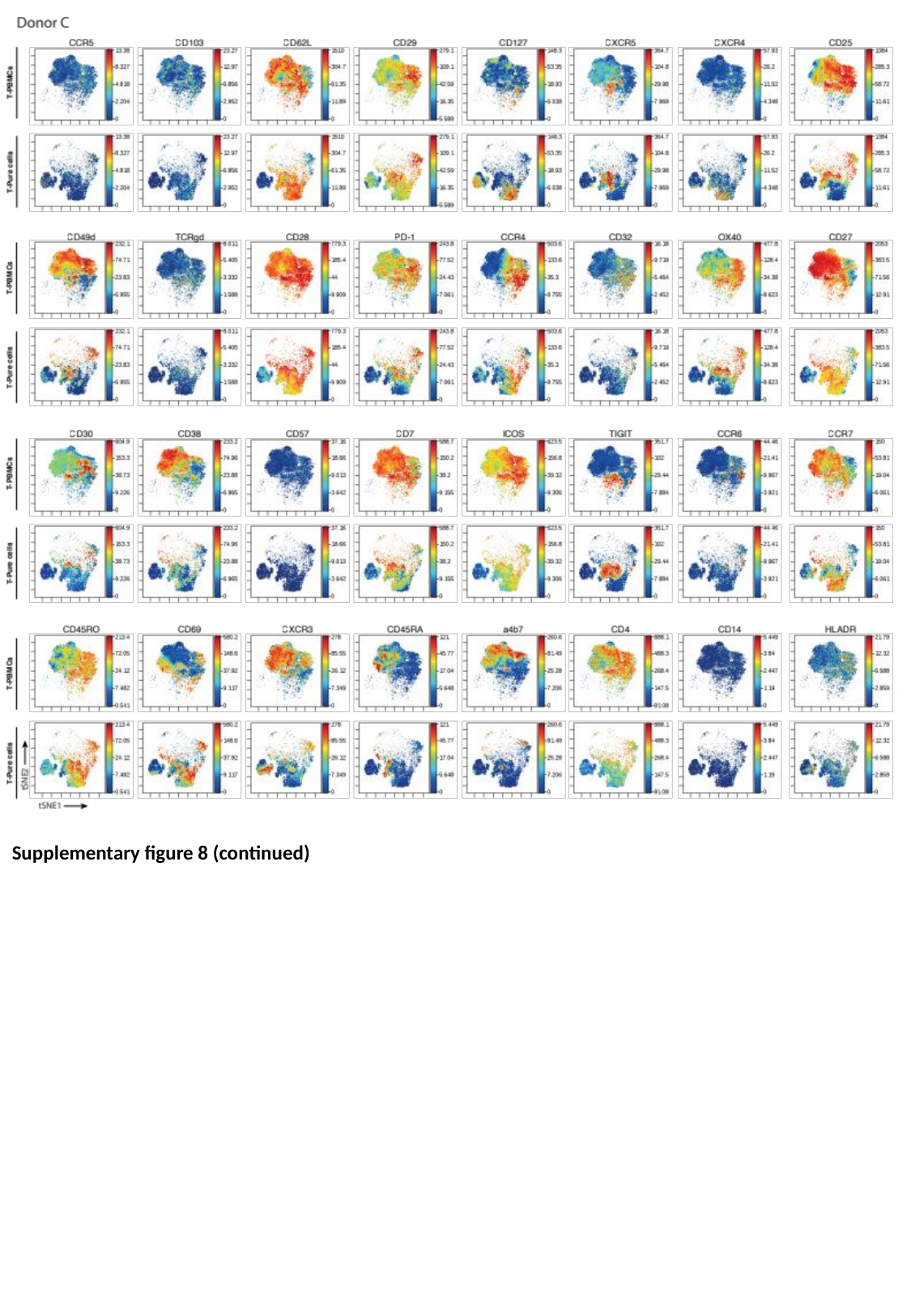

Supplementary figure 8 (continued)

## Slide 11
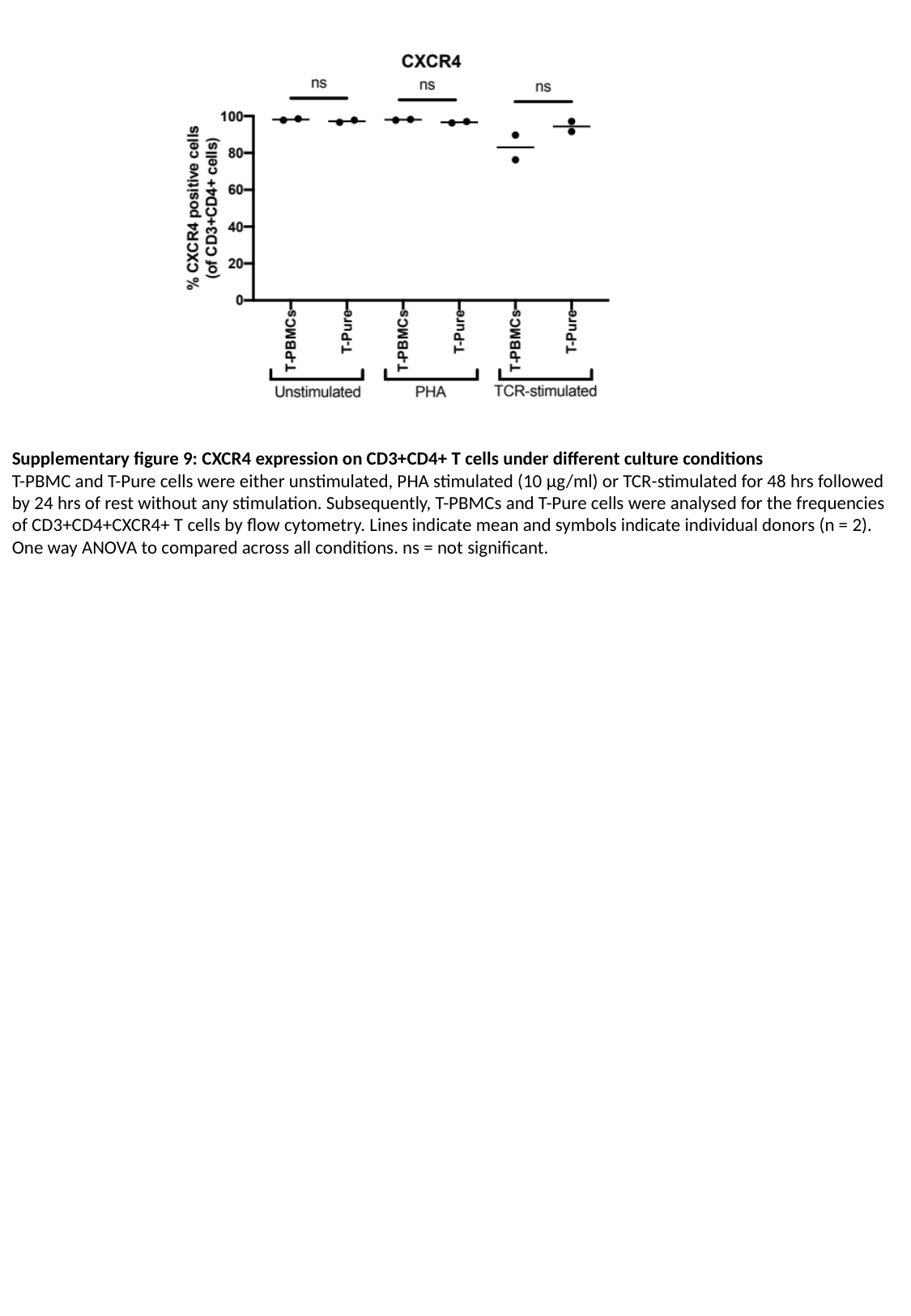

Supplementary figure 9: CXCR4 expression on CD3+CD4+ T cells under different culture conditions
T-PBMC and T-Pure cells were either unstimulated, PHA stimulated (10 µg/ml) or TCR-stimulated for 48 hrs followed by 24 hrs of rest without any stimulation. Subsequently, T-PBMCs and T-Pure cells were analysed for the frequencies of CD3+CD4+CXCR4+ T cells by flow cytometry. Lines indicate mean and symbols indicate individual donors (n = 2). One way ANOVA to compared across all conditions. ns = not significant.

## Slide 12
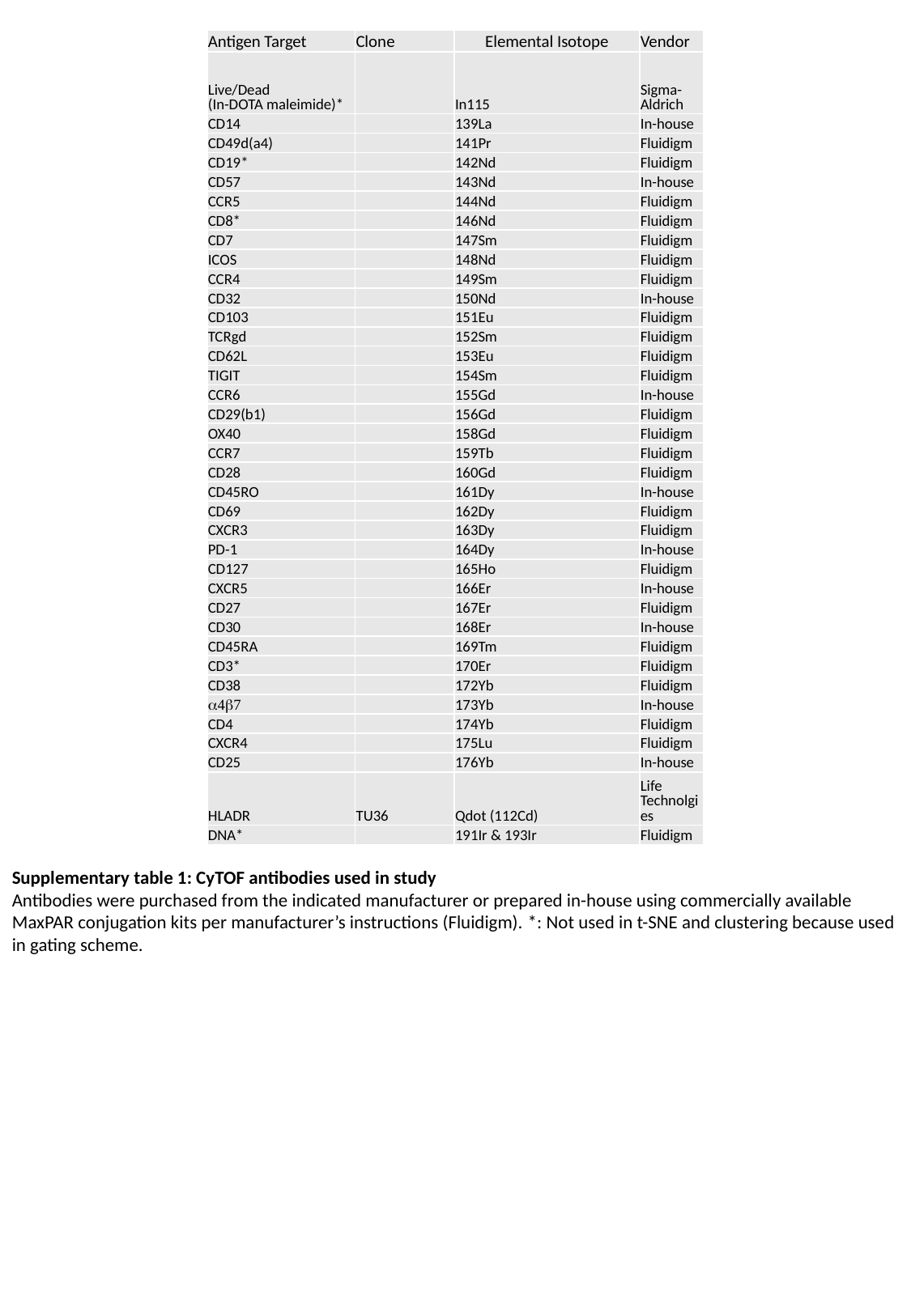

| Antigen Target | Clone | Elemental Isotope | Vendor |
| --- | --- | --- | --- |
| Live/Dead (In-DOTA maleimide)\* | | In115 | Sigma-Aldrich |
| CD14 | | 139La | In-house |
| CD49d(a4) | | 141Pr | Fluidigm |
| CD19\* | | 142Nd | Fluidigm |
| CD57 | | 143Nd | In-house |
| CCR5 | | 144Nd | Fluidigm |
| CD8\* | | 146Nd | Fluidigm |
| CD7 | | 147Sm | Fluidigm |
| ICOS | | 148Nd | Fluidigm |
| CCR4 | | 149Sm | Fluidigm |
| CD32 | | 150Nd | In-house |
| CD103 | | 151Eu | Fluidigm |
| TCRgd | | 152Sm | Fluidigm |
| CD62L | | 153Eu | Fluidigm |
| TIGIT | | 154Sm | Fluidigm |
| CCR6 | | 155Gd | In-house |
| CD29(b1) | | 156Gd | Fluidigm |
| OX40 | | 158Gd | Fluidigm |
| CCR7 | | 159Tb | Fluidigm |
| CD28 | | 160Gd | Fluidigm |
| CD45RO | | 161Dy | In-house |
| CD69 | | 162Dy | Fluidigm |
| CXCR3 | | 163Dy | Fluidigm |
| PD-1 | | 164Dy | In-house |
| CD127 | | 165Ho | Fluidigm |
| CXCR5 | | 166Er | In-house |
| CD27 | | 167Er | Fluidigm |
| CD30 | | 168Er | In-house |
| CD45RA | | 169Tm | Fluidigm |
| CD3\* | | 170Er | Fluidigm |
| CD38 | | 172Yb | Fluidigm |
| a4b7 | | 173Yb | In-house |
| CD4 | | 174Yb | Fluidigm |
| CXCR4 | | 175Lu | Fluidigm |
| CD25 | | 176Yb | In-house |
| HLADR | TU36 | Qdot (112Cd) | Life Technolgies |
| DNA\* | | 191Ir & 193Ir | Fluidigm |
Supplementary table 1: CyTOF antibodies used in study
Antibodies were purchased from the indicated manufacturer or prepared in-house using commercially available MaxPAR conjugation kits per manufacturer’s instructions (Fluidigm). *: Not used in t-SNE and clustering because used in gating scheme.
